# Effects of Cavity Thickness and Mold Surface Roughness on the Polymer Flow during Micro Injection Molding

**DOI:** 10.3390/polym15020326

**Published:** 2023-01-08

**Authors:** Jiquan Li, Haowei Ma, Wenyong Liu, Shaofei Jiang, Baisong Pan

**Affiliations:** 1 College of Mechanical Engineering, Zhejiang University of Technology, Hangzhou 310014, China; 2 National International Joint Research Center of Special Purpose Equipment and Advanced Processing Technology, Zhejiang University of Technology, Hangzhou 310014, China; 3 Taizhou Institute, Zhejiang University of Technology, Taizhou 318012, China

**Keywords:** micro injection molding, surface roughness, cavity thickness, polymer flow

## Abstract

In micro injection molding, the cavity thickness and surface roughness are the main effects factors of polymer flow in the die designing and affect the quality of molded products significantly. In this study, the effects of cavity thickness and roughness of cavity surface were investigated mainly on polymer flow during molding and on the roughness of molded products. The parts were molded in the cavities with the thickness from 0.05 mm to 0.25 mm and surface roughness from R_a_ = 46.55 nm to R_a_ = 462.57 nm, respectively. The filling integrities and roughness replication ratio of molded parts were used to evaluate the statements of polymer flow and microstructure replication during micro injection molding, respectively. The results showed that the filling integrity changing trends in the thinner cavities were obviously different or even opposite to those in the thicker cavities with the changing of cavity surface roughness instead of single trend in the conventional studies. For each cavity surface roughness, the filling integrity showed an upward trend with the increasing cavity thickness. In different cavity thickness, the maximum gap of filling integrity was 23.76 mm, reaching 544.94% from 0.05 mm to 0.25 mm. Additionally, the surface roughness ratio was slightly smaller than one before, reaching the polymer surface roughness limit around R_a_ = 71.27 nm, which was decided by the nature of the polymer itself. This study proposed the references for the design and fabrication of mold cavities and parts, and saved time and cost in the actual product manufacturing.

## 1. Introduction

Injection molding [1,2] is a critical molding method of thermoplastics and their composites [3] and it has been widely used. Micro injection molding (μIM) [4,5,6] is an injection molding process that has been used in manufacturing precision microscale plastic parts, such as automotive, micro biomedical and optical elements [7,8,9]. Unlike conventional injection molding (CIM) processes, the μIM processes are employed in small size and high precision situations [7,10]. Additionally, the improper manufacturing process and processd parameters will affect product quality [11]. The polymer flows with high speed and pressure in the cavities to guarantee the quality of the products, otherwise, there will be varieties of defects [12]. However, there are many intractability problems to be solved in μIM [13], especially the polymer flow during molding, which mainly includes two aspects: insufficient filling and incomplete microstructure replication [14,15]. Both of the aspects are mainly affected by process parameters [16,17], cavity thickness [18] and surface roughness [19]. Among these factors, the cavity thickness and surface roughness are not only more difficult to adjust, but are often constrained by product requirements.

Many researchers have investigated the effects of cavity surface roughness on insufficient filling [20,21]. Some of them believed that the cavity surface roughness promoted the polymer flow in the cavity [22,23]. The phenomenon was explained by wall slip phenomena and heat transfer theories. Researchers believed that the micromorphology of the cavity surface can promote wall slip and thus increase the filling length [24]. On the other hand, the surface roughness of the cavity affected the weld mark of the product surface and indirectly affected the tensile strength [25]. The heat transfer coefficient (HTC) increased with the increasing of cavity surface roughness, which helped the polymer flow [26]. However, some other researchers believed that the cavity surface roughness hindered the polymer flow [27] or had no effects on the polymer [28] flow in the cavity. The reason for the contradiction was mainly due to the difference in cavity size, in particular, the thickness of the cavity. Therefore, these conclusions were not comprehensive, and further study was required to draw more systematic and comprehensive conclusions.

The effect of the cavity size on the micro injection molding process was different, and the effect law was different from the CIM case [29]. Wang et al. [16] investigated the effect of cavity size on the product microstructure. Three different morphologies were observed in the microstructure of products with thicknesses of 1 mm, 200 μm and 100 μm. Xu et al. [30] studied the difference between micro and macro scales, and experimentally investigated the viscous dissipation of melt in channels with the same diameter ratio of 1 mm, 500 μm and 350 μm. The viscous dissipation effects of molten polymers in microscale cavities were different from those in conventional cavities. All the references showed that the cavity size affected the polymer flow significantly and that the microscopic differences in the cavities were non-negligible.

It also attracted the attention of researchers to the replication of product surface roughness to cavity surface roughness as a kind of microstructural replication [26,31]. Many researchers believed that the parts cavity roughness was smaller than cavity surface roughness [32,33]. They believed that the air tripping and the incomplete milling marks replicating were the reasons for surface roughness changes. Additionally, the grove part also had a certain amount of air, resulting in the peak of the product always being lower than the depth of the groove on the surface of the cavity, so that the roughness became smaller [22]. Whereas, other researchers reached the conclusion that the part surface roughness was larger than the cavity surface roughness in CIM [34] as the cavity surface roughness was small. Due to the surface tension and viscosity of the polymer, the molten polymer cannot enter the fine structure of the microstructure of the surface, resulting in a low replication level. However, in μIM, the relationship of surface roughness between cavities and parts was different because the cavity size was small and the polymer flow rules is different from CIM. As a result, it was necessary to investigate the surface roughness replicating rules between cavities and parts.

According to the studies above, the investigation of polymer flow during micro injection molding should consider the cavity thickness and surface roughness at same time. As a result, in this study, the effects of the cavity surface roughness and the cavity thickness on polymer flow were investigated mainly on the polymer flow during molding and on the roughness of molded products. A wider range of cavity thickness and surface roughness was introduced, which enabled more comprehensive study results. The filling integrity and surface roughness replication ratio were proposed to evaluate the filling state in different thickness and surface roughness cavity. In addition, the parts were molded in the cavities with five different values of cavity thickness and cavity surface roughness. The results showed that the cavity surface roughness had different effects with the change of cavity thickness. When the cavity was thin, the effects of wall slip and viscosity dissipation affected the filling integrity significantly. Moreover, the parts surface roughness was not only affected by the cavity surface roughness, but also by the nature of the polymer itself. After this study, the effect of the process parameters on the polymer flow and the influence law should be investigated and the micro injection molding process window can be broadened. This study analyzed the molding rules in different cavity thicknesses and surface roughnesses in μIM and provided the references of cavity design.

## 2. Materials and Methods

### 2.1. Experiment

**Figure 1:**
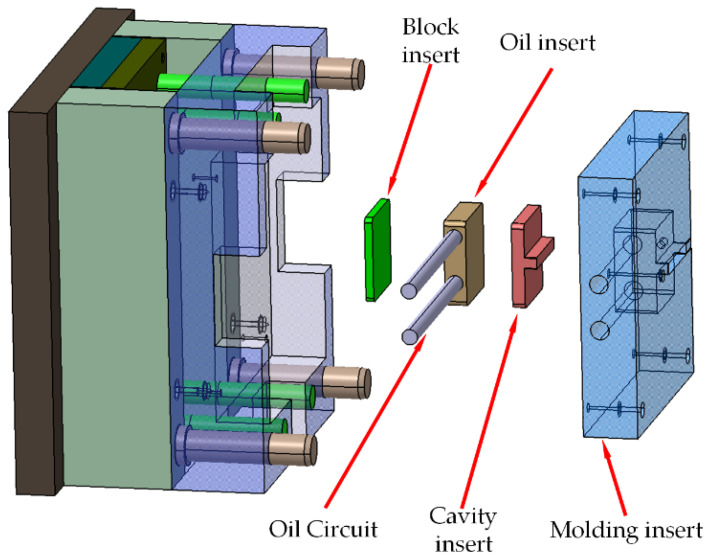
Schematic diagram of die assembly.

**Figure 2:**
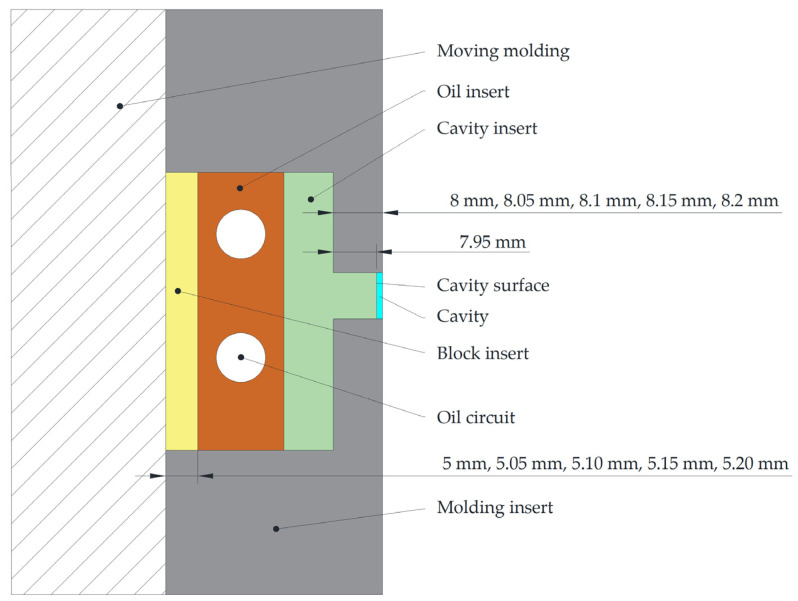
The specific assembly scheme of the die.

In this study, various combinations of insertions were used to adjust the cavity thickness and change the cavity surface roughness. The inserts were assembled in a molding insert on the moving die, which was composed by a block insert, an oil insert and a cavity insert, and was the assembled as shown in Figure 1. It was assembled into a cavity of variable thickness and surface roughness. The cavity thickness was changed by matching the cavity inserts of different sizes to the molding inserts. Additionally, the cavity surface roughness was adjusted by replacing the cavity inserts with different roughness surfaces. The specific assembly scheme was shown in Figure 2. The molding insert was 170 mm × 160 mm × 35.2 mm. The five heights of the molding inserts were 8 mm, 8.05 mm, 8.10 mm, 8.15 mm and 8.20 mm, respectively, on fit positions with the cavity inserts. The boss height of the cavity inserts was fixed at 7.95 mm, in which the boss is the cavity surface of the experimental cavities. The molding inserts with different boss heights matched with the fixed-size cavity insert. The bosses of the cavity inserts were polished by different polish method for different cavity surface roughness.

The raised parts of the block inserts were milled to five heights so that the cavities thicknesses were 0.05 mm, 0.10 mm, 0.15 mm, 0.20 mm and 0.25 mm. Additionally, the cavity inserts were polished using different polishing methods to measure the surface roughness of different cavities, which were shown in Table 1. The upper surfaces of the cavity inserts were designed as 7.5 mm × 50 mm rectangle [35], where the polymer will not fill the cavities completely to ensure that the polymer can reach its maximum filling capacity.

The parts were molded using polymethyl methacrylate (PMMA, HT55X, Sumitomo Chemical Co. Ltd., Tokyo, Japan), of which the MFR and Vicat Softening Temperature are 2 g/10 min and 113 °C, respectively. They was produced using a high-speed injection molding machine (VE4009(2)-80h-B, Ningbo Zhafir Plastics Machinery Manufacturing Co., LTD., Ningbo, China). The parameters of the process are listed in Table 2 based on previous experiments of our group during the study, and we chose the best polymer flow statement for next investigation to ensure the maximum filling capacity. In order to avoid fabrication errors during the experiments, the first five parts in each insertion combination were not used in the subsequent analysis. After excluding the first five parts, three more parts were formed for each cavity combination for subsequent experimental analysis.

### 2.2. Surface Roughness Measurement

The surface roughness of parts and cavities were using a nano 3D optical surface profilometer (SuperView, Chotest Technology Inc., Shenzhen, China). In this study, it was assumed that the cavity surface roughness was the same at any position of the cavity surface. Three points on the cavities were chosen to determine the roughness of the cavities, which were named point A, B as well as C and located on left, middle and right of the cavities, respectively, as shown in Figure 2. In order to investigate the roughness replication ratio, beside measuring the cavities surface roughness, it was also necessary to measure the surface roughness of the parts. The measurement point D on the part was the point where all the parts in the experiment can be filled. Additionally, it was the same position as the points on the cavities for the sake of simplicity. The position of the point D on the parts is shown in Figure 2. For more stable experimental results, the surface roughness of each point was measured three times, and the average value was taken as the surface roughness of the point.

### 2.3. Evaluation of Polymer Flow

In this study, the evaluation of polymer flow was divided into macroscopic and microscopic aspects. In macroscopic aspect, because of the intuitive of filling integrity, it was the most common indicators [22,36]. In another aspect, the roughness replication ratio was used to evaluate the microcosm filling.

#### 2.3.1. Parts Filling Integrity

The filling integrity was calculated by the ratio of parts acreage to cavities acreage, and the ratio was calculated by Equation (1):

(1) $$L\;=\;(S_{1}/S_{2})\times l,$$

where *L* (mm) is the ratio of filling acreages. In addition, *S*_1_ (mm^2^), *S*_2_ (mm^2^) and *l* (mm) are parts acreage, cavities acreage and cavities length along the flow direction, respectively, and in this study, *l* = 50 mm. 

**Figure 3:**
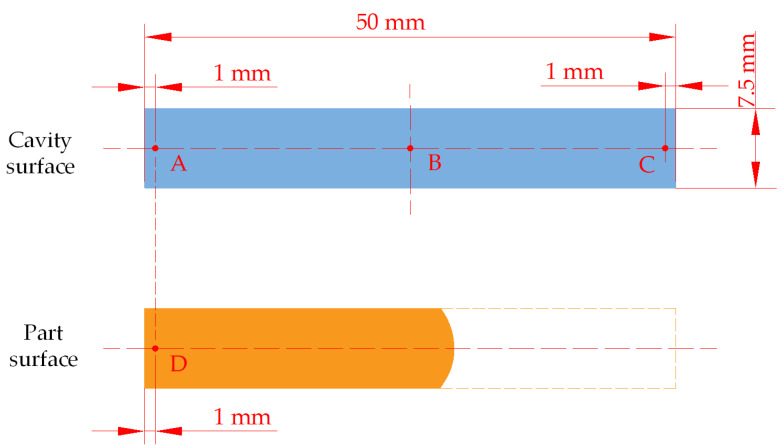
Measuring point of cavities surface roughness.

To determine the parts acreage and cavities acreage, an image processing method [37] was introduced in this study and it is shown in Figure 3. First, a standard white rectangle, same as the cavities, was printed on black paper at a ratio of 1:1, with a large color difference from the background, so that the cavities’ contours could be easily identified at a later stage. The parts were placed next to the cavities pattern, which has a large difference between its color and the background color, and the contour of the part can be easily identified by image processing to calculate its acreage. The standard cavities’ contour and the parts were photographed, and then the images were processed to obtain the standard cavity contour images and the parts images. The acreage of the parts and cavities were calculated by the number of pixels contained in the standard cavities contour image and the parts image. It was supposed that the *n*_1_ (pt) and *n*_2_ (pt) were the number of pixels in parts image and standard cavities image, according to Equation (1), the filling integrity was deduced below as Equation (2).

(2) $$L=50n_{1}/n_{2}$$

#### 2.3.2. Roughness Replication Ratio

The roughness replication ratio was calculated from the surface roughness of the parts and cavities. It is worth noting that the roughness replication ratio is the ratio of the cavities to the same position on the cavities, since the melt polymer cannot completely fill the cavities. In this study, it was the ratio of surface roughness of point D and A in Figure 2. The calculation method is shown in Equation (3) where *R_ap_* (nm) and *R_ac_* (nm) were the contour arithmetic average deviation (R_a_, nm) of the parts and cavity, respectively. *R_qp_* (nm), *R_qc_* (nm), *R_zp_* (nm) and *R_zc_* (nm) were the contour root mean square deviation (R_q_, nm) and ten points height of microcosmic unflattens (R_z_, nm) of the parts and cavity, respectively. *Q_a_* (-), *Q_q_* (-) and *Q_z_* (-) were the roughness replication ratio of R_a_, R_q_ and R_z_, respectively.

(3) $$\left\{ \begin{array}{l} Q_{a}=R_{ap}/R_{ac}\\ Q_{q}=R_{qp}/R_{qc}\\ Q_{z}=R_{zp}/R_{zc} \end{array}\right.$$

### 2.4. Full Factorial Experimental Design

In this study, the full factorial experimental method was introduced to investigate the effects of each assembly strategy on filling for the combined effects of factors. The cavities had 25 combinations of thickness and roughness depending on the different assemble strategies. The cavity thickness and surface roughness were taken as variables, where the surface roughness of cavities was marked as R_1_, R_2_, R_3_, R_4_ and R_5_ with the improvement of it.

## 3. Results and Discussion

### 3.1. Effects of Cavity Surface Roughness on Filling Integrity with Single Cavity Thickness

**Figure 4:**
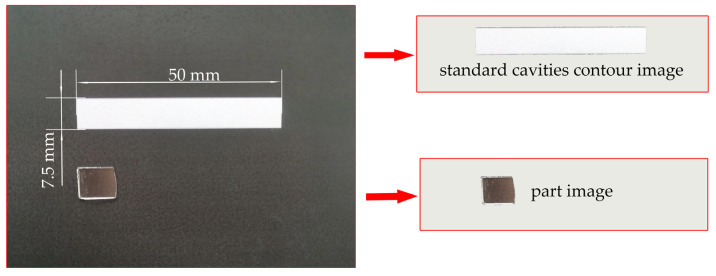
Schematic diagram of image processing.

The surface roughness and topography of the cavity are shown in Table 3 and Figure 4, respectively. The surface roughness of the cavities was the mean value of the surface roughness of points A, B and C.

**Figure 5:**
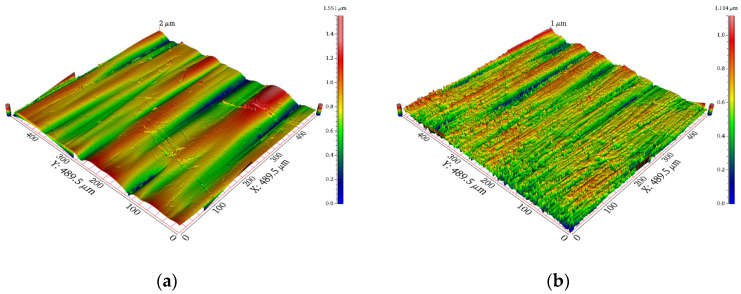

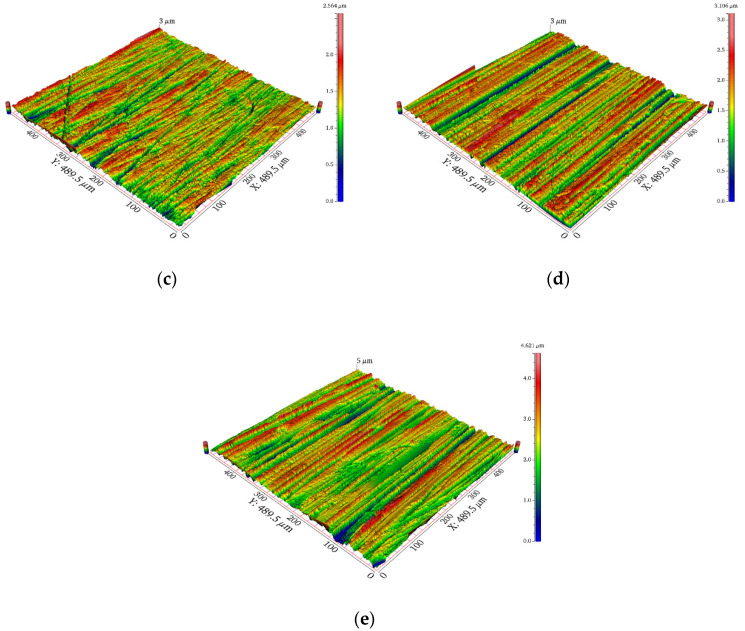
The topography of each cavity surfaces: (**a**) R_1_, (**b**) R_2_, (**c**) R_3_, (**d**) R_4_ and (**e**) R_5_.

For the five different cavity thicknesses, the experimental results for filling integrity are shown in Figure 5. For a cavity thickness of 0.05 mm, the filling integrity of the parts first increased, then decreased with increasing surface roughness of the cavity, and then increased sharply at R_5_. For cavity thicknesses of 0.10 mm, 0.15 mm and 0.20 mm, the effect of the surface roughness of the cavity on filling integrity was similar, with the filling integrity of the parts first increasing and then decreasing as the roughness of cavity surface increased. The inflection points for different cavity thicknesses were obviously different. For the cavity thicknesses of 0.10 mm and 0.15 mm, the inflection point was R_4_, while for the 0.20 mm, the inflection point was R_3_. However, the cavity thickness of 0.25 mm was different from the previous results. The filling integrity at the 0.25 mm cavity first decreased and then increased with increasing roughness of cavity surface.

In general, the increasing of the surface roughness of the cavity increased the total surface area of the molten polymer in contact with the cavity surfaces, which led to an increase in heat transfer coefficient. Additionally, the filling integrity increased with the increase of the roughness of the cavity surface. However, in very thin cavities with high surface roughness, the cortical polymers may interact with adjacent polymer layers to generate shear stress. The frictional force between molten polymer and the cavity surfaces was counteracted by this phenomenon, resulting in wall slip being generated and increasing the filling integrity of the molten polymer in the cavity.

Additionally, the viscous dissipation was promoted as the shear rate of polymer increased, which was inferred that the viscosity of the polymer decreases. Moreover, the cavity temperature was increased by the high shear rate that promoted the filling of polymer and the filling integrity was increased. On the other hand, the effects of microscopic grooves on the cavity on filling were more pronounced as the surface roughness of the cavity was improved. Due to the uneven filling of the molten polymer, the microscopic grooves were not filling completely as the roughness of the cavity surface increased. There was a layer of air between the molten polymer and the surface of the cavity, which caused the contact area and the solidification rate to decrease. In this case, the combined effects of the high roughness of the cavity surfaces were to facilitate the flow of the molten polymer in the cavity.

The effects of the roughness of the cavity surface on the filling had a critical point, which varied with the cavity thickness. When the cavity was very thin and the cavity surface roughness smaller than R_3_, such as 0.05 mm, the filling integrity improved with the increase of cavity surface roughness. This showed that the effect of cavity surface roughness on filling is greater than its inhibition. However, the suppression was larger when the cavity surface roughness was larger than R_3_. When the cavity surface roughness was R_5_, the filling integrity increased sharply. In other cavity thickness, the critical points were also shown. However, there was only one critical point. This demonstrated that there may be more than one critical point during the filling process, especially if the cavity is very thin.

### 3.2. The Difference of Filling Integrity of Different Cavity Surface Roughness with Different Thicknesses

**Figure 6:**
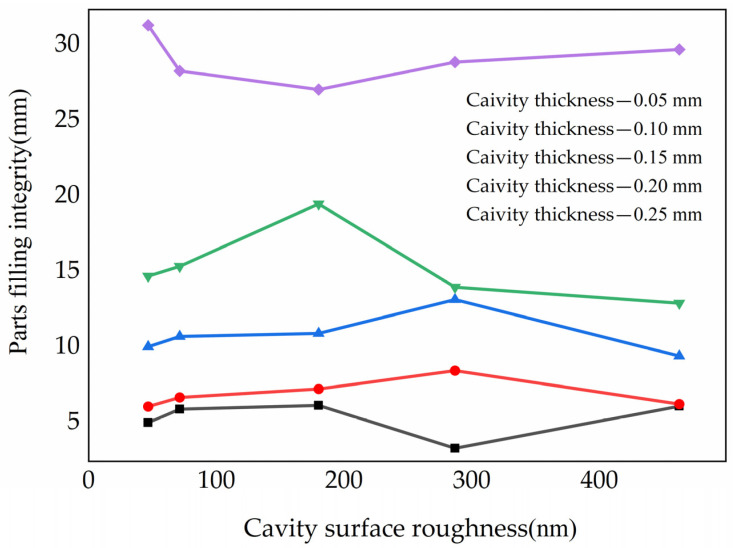
The filling integrity under a certain cavity thickness.

For better understanding the effects of the cavity surface roughness on the filling integrity, the maximum gaps of filling integrity with different cavity surface roughness were investigated. The analysis of the maximum gap of filling integrity was performed by calculating the difference of the maximum filling integrity within a certain cavity thickness minus the minimum one and dividing by the minimum one, and the results are shown in Figure 6. The maximum difference of filling integrity in the figure was the mean filling integrity for different cavity surface roughness. It can be deduced that the maximum gap of filling integrity, which was influenced by the roughness of the cavity surface, was different for different cavity thicknesses. When the cavity thickness was 0.05 mm, the maximum difference filling integrity among different parts was 85.07%. However, for the cavity thickness of 0.10 mm and 0.15 mm, the maximum difference was around 40% of the similarity. Moreover, when the cavity thickness was 0.20 mm, the maximum gap rose slightly. After the condition with cavity thickness of 0.20 mm, the maximum gap decreased sharply. The maximum gap was reduced to 15.68% when the cavity thickness was 0.25 mm.

It was found that the effects of the roughness of the cavity roughness on the filling flow of the molten polymer gradually decreased with increasing cavity thickness, as shown in Figure 6. The effects that named as microscale effect generated because of the μIM were different from CIM in principle and regularity [29]. Additionally, the effects also explained the contradiction in the relationship between the roughness of the cavity surface and the cavity thickness. Cavity thickness influenced the filling laws significantly during the injection process and deduced different results.

### 3.3. The Effects of Cavity Thickness on Filling Integrity

**Figure 7:**
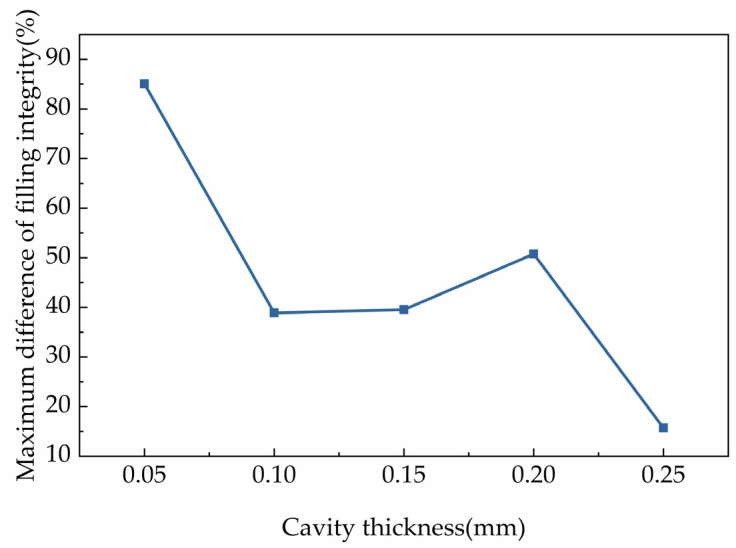
Maximum difference of filling integrity with different cavity thicknesses.

**Figure 8:**
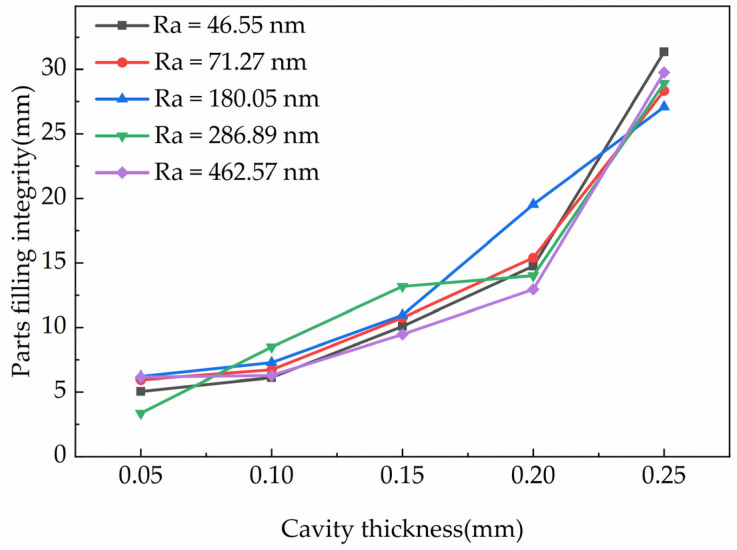
Parts filling integrity in cavities with different cavity thicknesses.

The cavity thickness was an important factor on filling integrity. In order to better understand the effects of the cavity thickness on the filling integrity, the changing trend of filling integrity within different cavity thickness was investigated. Figure 7 showed the changing trend of filling integrity in cavities with different cavity thickness. It can be inferred in the figure that the filling integrity and the rate of improvement were improved as the increasing of the cavity thickness. Moreover, in Figure 8, it showed the parts filling integrity in cavities with different cavity thicknesses. It can be inferred that the average filling integrity was also improved with the increasing of the cavity thickness.

According to Figure 7 and Figure 8, it was deduced that the effect trends of the cavity thickness on the filling integrity was basically the same. The filling integrity improved with the increasing of the cavity thickness and the slope increased gradually. When the cavity thickness increased from 0.05 mm to 0.25 mm, the filling integrity increased from 5.34 mm to 29.10 mm. In thinner cavities, when the cavity thickness was changed, there was a large change in the filling integrity of the parts. This phenomenon illustrated that the ratio of the wall slip length to the velocity of the molten polymer decreased with the increase of the cavity thickness [38]. This meant that increasing the cavity thickness weakens the effects of wall slip. When the relative surface area of the cavity increased, the convective heat transfer rate of the molten polymer increased. The molten polymer was rapidly cooled and the polymer flow was suppressed in the cavities. The effects of the cavity thickness on the filling integrity were much larger than the wall slip, leading to a sharp improvement in the filling integrity with the increase of cavity thickness.

### 3.4. The Effects of Cavity Surface Roughness on Roughness Replication Ratio

**Figure 9:**
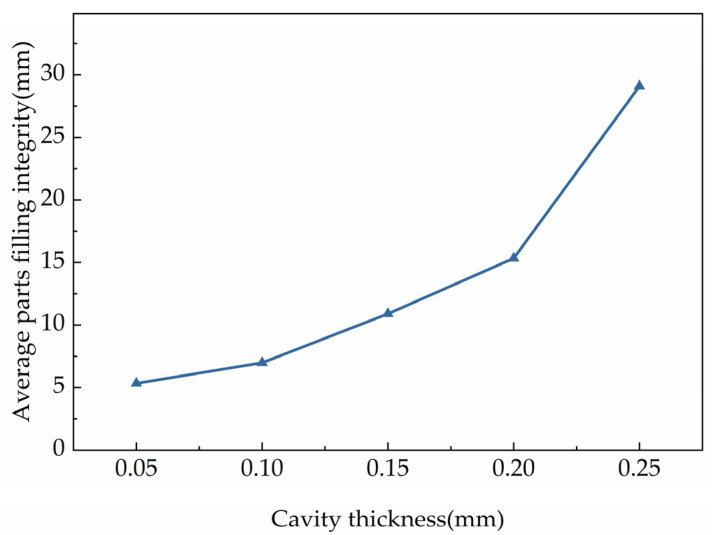
Average parts filling integrity with cavities with different thickness.

**Figure 10:**
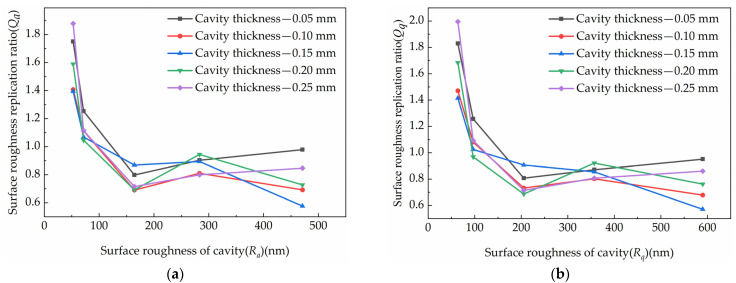

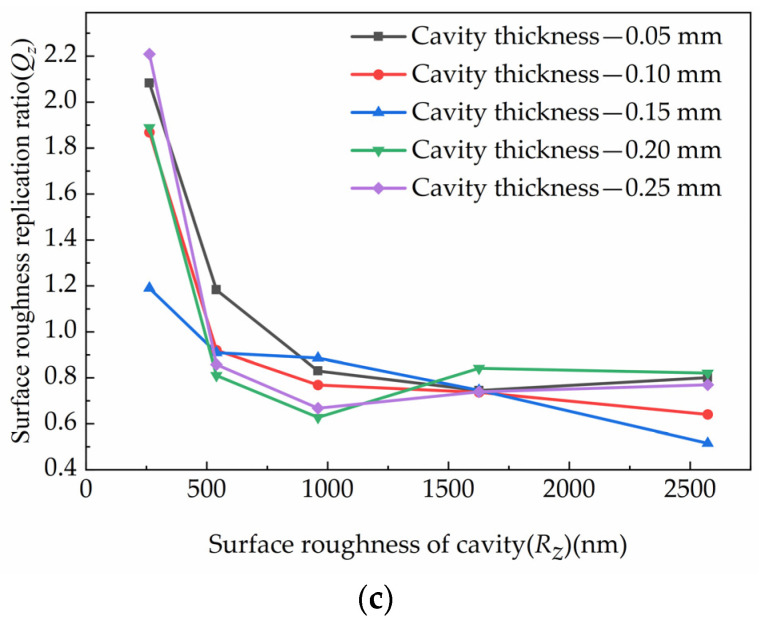
The replication ratio of each roughness parameter under different cavity thickness: (**a**) *Q_a_*, (**b**) *Q_q_* (**c**) *Q_z_*.

The micro-structure replication was seen as the polymer flow in a micro scale. Afterwards, the cavity surface roughness was considered as an irregularly distributed micro-structure on the surface of cavities. Additionally, the molten polymer filling was the process that replicated the micro-structures on the cavity. In this study, the roughness replication ratio was used to investigate the replication process. The roughness replication ratio of the left section was the ratio of A to D. Figure 9 showed the average filling integrity was shown to vary with different cavity thicknesses. Moreover, the replication ratio of each roughness parameter in different cavity thicknesses was shown in Figure 10.

The replication ratio trends were similar in different methods of surface roughness characterization. When the roughness of the cavity surface was relatively large, all three kinds of roughness replication ratio were less than one. This meant that the parts surface roughness was smaller than the cavity surface roughness. This conclusion demonstrated that the molten polymer did not fill the micro-structures on the cavities completely. The flow properties of the polymer were poor and the micro-structures on the cavity were not replicated by the polymer completely. The micro-structures on the cavities surface were not replicated completely on the surface of the parts, and the whole surface of the product has a smaller micromorphology than the cavity surface. However, the opposite conclusion holds when the roughness of the cavity surface was relatively small. The minimum surface roughness of the parts was around R_a_ = 71.27 nm, which was larger than the surface roughness of the cavities. This was due to the large surface tension and viscosity of the polymer chains, which made it difficult for the molten polymer to enter the micro-structures on the surfaces of the cavity. The micro-structures of the part surfaces were not constrained by that of the cavity surfaces. The polymer chains were free to contract on the cavity surfaces, and the parts had a larger surface roughness after cooling.

## 4. Conclusions

This study investigated the effects of cavity thickness and surface roughness on filling in μIM and explained the reasons for the effects. Cavities with adjustable thickness and surface roughness were designed to investigate the effects of thickness and surface roughness. Filling integrity and roughness replication ratio were used to access the effects of different cavity surface roughness and thickness. The following conclusions can be drawn. (1) The effects of cavity surface roughness influences on filling were significant during the injection process, and the influence law was different with the changing of cavity thickness. (2) In a thin cavity, the viscous dissipation and wall slip might be more obvious with the cavity surface roughness improve. The filling integrity increased as a result of these microscopic phenomena. (3) The effects of cavity thickness were significant, and the maximum gap of filling integrity was 23.76 mm, reaching 544.94% from 0.05 mm to 0.25 mm. (4) The parts surface roughness was not only influenced by cavity surface roughness, but by the nature of the polymer itself. Additionally, the critical point was around R_a_ = 71.27 nm.

The similarities with results of other studies were that the cavity thickness and surface roughness affected the polymer significantly. Moreover, the trend of the effect of polymer flow on the cavity thickness was similar to that found in other works. However, the effect laws for cavity thickness and surface roughness were different to those of other studies. There was a critical point at which the influence law was different when the factors reach it. Moreover, the wider size range of the die gave the investigation more comprehensive results. The limitation of our study was that the physical explanation of the polymer flow phenomenon lacked experimental validation. In general, the possible practical applications of the results included two aspects. Firstly, the surface roughness of the cavity and the range of the cavity size was increased to allow for a comprehensive and accurate conclusion, which gave a basis for the manufacturing of thin wall products. Secondly, the investigation of the flow law was a reference for setting the process parameters of the micro injection molding process, which can help to broaden the process window.

In future investigations, the effect of the process parameters on micro injection molding in different cavity thicknesses and surface roughness should be investigated. The mapping model of the impact of cavity thickness, cavity surface roughness and each process parameter on the evaluation metrics should be established. The impact of each process parameter on the evaluation metrics and the interplay between each factor should be analyzed.

## Figures and Tables

**Table 1:** The corresponding polishing method of each insert.

Cavity Insert Number	Polishing Method
1	None
2	Polished by 60 Cw abrasive paper
3	Polished by 400 Cw abrasive paper
4	Polished by 1500 Cw abrasive paper
5	Polished by polishing paste

**Table 2:** The molding process parameters.

Processing Parameters	Values
Melt temperature (°C)	255
Mold temperature (°C)	100
Injection speed (mm/s)	280
Injection pressure (MPa)	160
Holding pressure (MPa)	128
Holding time (s)	7
Cooling time (s)	35

**Table 3:** The surface roughness of each cavity surfaces.

Cavity Insert No.	*R_a_* (nm)	*R_a_* Stand Deviation (nm)	*R_q_* (nm)	*R_q_* Stand Deviation (nm)	*R_z_* (nm)	*R_z_* Stand Deviation (nm)
R1	46.55	12.42	88.68	43.98	370.08	173.03
R2	71.27	13.72	132.50	49.73	802.26	242.41
R3	180.05	21.03	227.06	21.01	1114.77	68.24
R4	286.89	31.33	363.26	29.88	1701.14	40.71
R5	462.57	101.80	591.31	135.36	2793.20	690.79

## Data Availability

Not applicable.
